# Na,K-ATPase mediated and cardiotonic induced signaling in health and disease

**DOI:** 10.3389/fphys.2025.1694027

**Published:** 2025-11-14

**Authors:** Jordan Trant, Jenna Beth Lowery, Pedro Morales-Sosa, Gustavo Blanco

**Affiliations:** Department of Cell Biology and Physiology and The Kidney Institute, University of Kansas Medical Center, Kansas City, KS, United States

**Keywords:** Na,K-ATPase isoforms, cardiotonic steroid, cardenolide, bufadienolide, ouabain, intracellular signaling

## Abstract

In the late 1950’s, Na,K-ATPase (NKA) was discovered as the active transport system that establishes and maintains the transmembrane Na^+^ and K^+^ gradients necessary for cell survival and function. Almost 70 years later, a novel unexpected function for NKA was unveiled, when it was shown that NKA has the amazing versatility of playing a role beyond its classical “ion pumping” function to also serve as the receptor and signal transducer for the effects of cardiotonic steroids (CTS) in cells. Since then, the field of NKA research expanded into a new dimension. The additional unexpected finding that CTS are commonly present in the body fluids of mammals inspired investigators to further study the CTS-induced and NKA-mediated pathway, its mechanisms of action, effects in cells, and importance to tissue and body physiology. Therefore, a vast amount of information has accumulated in recent years. In this article, we attempt to review the most current information available, focusing on the effects of CTS and NKA signaling in physiological and pathological states. We also discuss controversies, unsolved issues, and future directions of this fascinating area of research.

## Introduction

In addition to its classical role in ion transport, a novel function for NKA was unveiled when it was found that it also serves as a plasma membrane receptor and a signal transducer for the effect of cardiotonic steroids (CTS) in different cells and tissues. In this review, we discuss various aspects of this fascinating role of NKA and its importance in physiologic and pathologic states. This field of research has grown very rapidly in the last several years as investigators tried to understand the mechanisms of action and effects of CTS in different cells and tissues. The vast literature makes it challenging to cover all articles available. Therefore, we would like to suggest the reader to also visit a series of excellent reviews on this topic (Xie, 2001; Harwood and Yaqoob, 2005; Pierre and Xie, 2006; Aperia, 2007; Tian and Xie, 2008; Bagrov et al., 2009; Riganti et al., 2011; Silva and Soares-da-Silva, 2012; Aperia et al., 2016; Askari, 2019; Orlov et al., 2021; Blaustein and Hamlyn, 2024; Gao et al., 2025).

## Na,K-ATPase as an ion transporter

The Na,K-ATPase (NKA) was discovered in 1957 as the cell plasma membrane ion transport system that maintains the low Na^+^ and high K^+^ concentrations inside the cells compared to the extracellular medium (Skou, 1957). These transmembrane gradients are essential for the secondary transport of other ions, nutrients, and water in and out of the cell, maintenance of cell volume, and generation of the cell resting membrane potential, which allows for the development of action potentials in excitable tissues (Féraille and Doucet, 2001; Contreras et al., 2024).

NKA is composed of at least two major subunits, α and β and, in some tissues, these proteins are joined by a third subunit, which belong to a family of polypeptides that have the common consensus sequence Phe-X-Tyr-Asp or FXYD (Sweadner, 1989; Lingrel and Kuntzweiler, 1994; Kaplan, 2002; Jorgensen et al., 2003; Garty and Karlish, 2005; Geering, 2005; Clausen et al., 2017). NKA subunits assemble in a 1:1:1 stoichiometry, but these complexes can also oligomerize forming dimers or higher order molecular structures at the cell plasma membrane (Lopina, 2000; Taniguchi et al., 2001; Laughery et al., 2004; Martin, 2005; Lindzen et al., 2006; Barwe et al., 2012; Yoneda et al., 2016). The NKA α subunit, also called the catalytic subunit, is a multi-spanning membrane protein directly involved in the translocation of the ions across the cell membrane. It contains the binding sites for Na^+^ and K^+^ and harbors the site for ATP hydrolysis, which fuels ion active transport. In addition, the extracellular face of the α subunit holds the molecular pocket where cardiotonic steroids (CTS) dock (Andersen and Vilsen, 1995; Lingrel et al., 1998; Jorgensen et al., 2003; Dyla et al., 2020). The NKA β subunit is a single membrane pass glycosylated membrane protein necessary for stabilizing the folding of the NKA α subunit during synthesis and for the intracellular trafficking and delivery of the whole enzyme to the cell plasma membrane (Ackermann and Geering, 1990; Geering, 1990; Geering, 1991; Chow and Forte, 1995; Ueno et al., 1997; Rajasekaran et al., 2004; Vagin et al., 2007). In epithelia and the nervous system, the NKA β subunit has been shown to also function as an adhesion molecule that participates in cell-cell interaction (Müller-Husmann et al., 1993; Tokhtaeva et al., 2011; Cereijido et al., 2012; Vagin et al., 2012). The FXYD subunit is a small type I membrane polypeptide that comprises several members, the first of which was discovered in the kidney and called the γ subunit (Therien and Blostein, 2000). While not essential for NKA enzymatic activity, the FXYD polypeptide is a regulator of NKA activity (Geering et al., 2003; Sweadner et al., 2003; Yap et al., 2021).

In mammals, NKA is expressed as a series of isozymes, which result from the association of different molecular variants or isoforms of each of its subunits (Blanco and Mercer, 1998; Mobasheri et al., 2000). The NKA α subunit exists as four isoforms (NKA α1-4) which have a tissue-specific distribution and different functional properties. NKA α1 is expressed in all tissues and is the isoform that maintains the basal Na^+^ and K^+^ transmembrane gradients in the cell (Lingrel, 1992; Blanco, 2005; Matchkov and Krivoi, 2016). NKA α2 is expressed primarily in cardiac, smooth, and skeletal muscles where it is essential for the contractility of the myofibers (He et al., 2001; Dostanic et al., 2003; Shelly et al., 2004; Swift et al., 2007; Aronsen et al., 2013; McKenna et al., 2024). NKA α2 is also expressed in other tissues; in the brain NKA α2 is present in glia and contributes to K^+^ homeostasis and neurotransmitter release in the synaptic cleft (Moseley et al., 2003; Kawakami and Ikeda, 2006; Larsen et al., 2016). In the lung, NKA α2 is important for alveolar fluid clearance (Ridge et al., 2003). In adipose tissue (Coppi and Guidotti, 1997), NKA α2 contributes to insulin regulated K^+^ uptake. The NKA α3 isoform is primarily expressed in neurons, where it re-establishes cell membrane potential after depolarization (Dobretsov and Stimers, 2005; Holm et al., 2016). NKA α3 was also identified in the adult heart, contributing there to the electrical activity of the heart (Sweadner, 1989; Kaplan, 2002; Lingrel, 2010). Finally, NKA α4 is the NKA isoform with the most restricted pattern of expression, being limited to the male germ cells of the testis and sperm, where it is essential for male fertility (Woo et al., 1999; Blanco et al., 2000; Wagoner et al., 2005).

Regarding the NKA β isoforms, three different polypeptides were found (NKA β1-3) which also have a tissue-specific distribution. NKA β1 is ubiquitously expressed, NKA β2 is present in muscle and glia, and NKA β3 is found in the retina, liver, lung, and testes (Blanco and Mercer, 1998). All three β isoforms associate with the various α polypeptides in different combinations when expressed *in vitro*, though these interactions are more limited *in vivo* since they depend on the particular subset of isoforms expressed in each tissue (Eakle et al., 1994; Blanco et al., 1995a; Blanco et al., 1995b).

With respect to the FXYD polypeptides, seven different variants have been described in humans, which like the NKA α and β subunits are distributed in a tissue-specific manner. While FXYD polypeptides have been shown to modulate the ion affinity and maximal activity of NKA, their functions *in vivo* are not yet well characterized (Yap et al., 2021).

As an ion transporter, NKA continuously cycles between two different main conformations: the E1 state (where in the presence of Na^+^ and ATP the NKA α subunit becomes phosphorylated) and the E2 state (where in the presence of K^+^ and ATP the NKA α subunit dephosphorylates) (Glynn, 1956; Apell et al., 1998; Kaplan, 2002; Martin, 2005). In each pumping cycle, NKA moves 3 Na^+^ out and 2 K^+^ into the cell, while one molecule of ATP is hydrolyzed (Glynn, 1956; Post and Jolly, 1957). Under the physiological environment of the cell, the Na^+^ and ATP levels are critical for the regulation of NKA activity (Bertorello and Katz, 1993; Yu, 2003). Several hormones also regulate NKA activity by mechanisms that include: 1) changes in the activity of preexisting NKA molecules at the cell membrane; 2) modification of the expression of NKA molecules through changes in gene transcription and translation (Horowitz et al., 1990; Orlowski and Lingrel, 1990; Féraille and Doucet, 2001); or 3) post-translational phosphorylation of the α subunit, which regulates NKA insertion and retrieval from the cell plasma membrane (McDonough and Farley, 1993; Ewart and Klip, 1995; Gao et al., 1999; Therien and Blostein, 2000; Figtree et al., 2009; Poulsen et al., 2010; Cordeiro et al., 2024).

## Na,K-ATPase as a receptor and signal transducer

The initial observation that NKA had effects that go beyond its function of maintaining the transmembrane gradient for Na^+^ and K^+^ in the cell was suggested by experiments performed in the early 1970s, which showed that chronic treatment of cultured cells with ouabain upregulates the expression of the genes that encode for the NKA α and β subunits (Pressley, 1988). This intriguing finding suggested that NKA could somehow communicate with the cell nucleus to regulate gene transcription and protein synthesis through induction of early response proto-oncogenes and activation of transcription factors. Further work expanded the list of genes that were regulated by ouabain and showed that this compound also enhanced cell growth. It was also realized that those effects were separate from changes in intracellular ions due to NKA inhibition (Xie and Askari, 2002).

**FIGURE 1:**
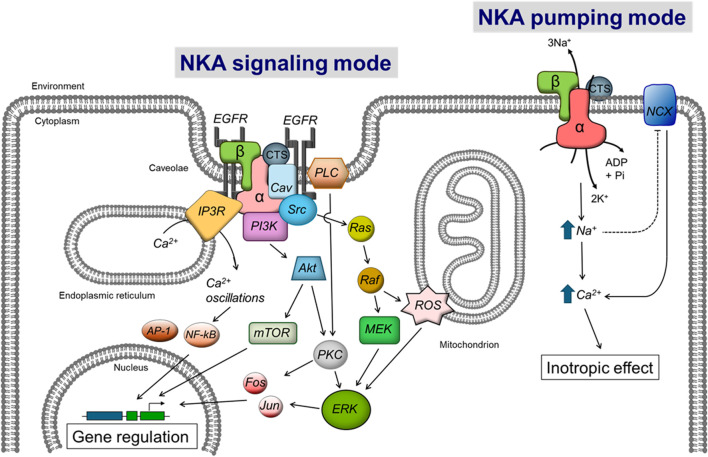
Scheme showing the control of cell function by CTS via the NKA ion transport and signaling functions. The right side of the diagram shows the Inhibition of NKA pumping mode by CTS, which directly raises intracellular Na^+^ levels. This in turn secondarily augments cytosolic Ca^2+^ via the Na/Ca exchanger (NCX) to elicit the “cardiotonic effect”. The left side of the figure shows the activation of NKA signaling by CTS, which stimulates a series of downstream intracellular intermediates that will ultimately modulate gene expression. (CTS) cardiotonic steroid, (α and β) subunits of the Na-K-ATPase. See the text for additional definitions.

The modulatory effects of ouabain on gene expression went dormant for some time until the early 1990s, when studies spearheaded by Askari and Xie showed that ouabain treatment of neonatal myocardial cells in culture activated downstream pathways in the cells and activated gene transcription in a fashion that resembled the effect of growth factors and hormones (Peng et al., 1996; Huang et al., 1997; Xie et al., 1999; Haas et al., 2000; Aizman et al., 2001; Xie and Askari, 2002; Miyakawa-Naito et al., 2003; Khundmiri et al., 2006). CTS modulate gene expression in tissues different than the heart; for example, using differential display analysis to compare mRNA levels, it was shown that ouabain, bufalin and norbufalin downregulate expression of the signalin protein 14-3-3 in rat lens (McGowan et al., 1999). These results represented the starting point of what we know today as the “non-pumping” or “signaling” function of NKA. In this way, the concept evolved that ouabain is on the one hand an inhibitor of NKA transport activity, which modifies ion concentrations inside the cell; and on the other, an activator of NKA mediated signal transduction, which modulates gene and protein expression. Both events, shown in Figure 1, play a critical role in overall cell function. The fascinating discovery of a downstream signaling pathway activated by CTS gave a new dimension to the role of NKA in cells and opened new unexpected avenues of research for this ion transporter.

## Cardiotonic steroids; the ligands that trigger NKA signaling

CTS include two main types of structures: cardenolides, of which ouabain, digoxin, and digitalis are the most widely studied members (Schoner and Scheiner-Bobis, 2007b; Botelho et al., 2019; Patocka et al., 2020), and bufadienolides, which have bufalin and marinobufagenin as the main representative compounds (Bagrov et al., 2009; Agrawal et al., 2012; Asrorov et al., 2023). Structurally, these chemical scaffolds consist of 1) a steroidal backbone, with A/B and C/D rings in a cis conformation; 2) a lactone ring attached to C17, which in cardenolides is a five-member unsaturated butyrolactone and in bufadienolides a six-member pyrone ring; and 3) a sugar moiety at C3, which is highly variable depending on the CTS considered (Botelho et al., 2019). A characteristic of CTS is their vast natural chemical diversity, with important differences in their steroid skeleton, lactone moiety and sugar group. The best-known naturally occurring CTS include the cardenolides ouabain, digoxin, digitoxin, and oleandrin; and the bufadienolides bufalin, hellebrin, and marinobufagenin.

Ouabain was one of the first CTS found to have a practical application. Initially, it was used in low amounts as herbal remedies and in high concentrations as a poison (Goldman, 2001; Wachtel-Galor and Benzie, 2011). In the laboratory setting due to its hydrophilic property and high specificity, ouabain has been classically employed to distinguish NKA ion transport and activity from the function of other ATPases. Ouabain-NKA interaction requires the presence of Na^+^ and ATP and is antagonized by K^+^ (Schultheis et al., 1993). Once bound, ouabain interferes with NKA reaction cycle, locking the enzyme in the E2 conformation (Hansen, 1982).

When used at relatively high concentrations, ouabain and other CTS target and inhibit NKA activity. Digitalis and digoxin have found important application in modern medicine, inducing a beneficial increase in cardiac inotropy, chronotropy, and hypertrophy, which have made them useful agents for the treatment of congestive heart failure and atrial fibrillation (Ehle et al., 2011; Sethi et al., 2018; Whayne, 2018; Meijler, 1985; Bessen, 1986). These CTS, however, have a different therapeutic profile than ouabain (Fuerstenwerth, 2014). Despite their widespread clinical usage, the mechanisms of action of CTS remained elusive for decades. It is now known that the inhibition of NKA activity by CTS causes a primary increase in intracellular Na^+^ ([Na^+^]_i_) which, through the Na/Ca exchanger, secondarily raises Ca^2+^ concentrations in the cardiac cells (Blaustein et al., 2016). This increase in cytosolic Ca^2+^ favors heart contractility, the main effect for which CTS have received their distinct name. In addition to increasing heart contractility through partial inhibition of NKA ion transport, CTS can also exert this effect via NKA signaling (Buzaglo et al., 2016; Buzaglo et al., 2019). Both mechanisms of action of CTS effects mediated by NKA ion transport and signaling are shown in Figure 1. The cardiotonic effect is thought to be mediated by the NKA α2 isoform, highly expressed in the cardiac T-tubules, whereby it functionally interacts with the Na/Ca exchanger. While excellent as positive inotropic agents, the clinical use of CTS declined over the years due to their narrow therapeutic window and adverse effects (Patocka et al., 2020).

Originally, it was thought that CTS were only the products of plants and amphibians and that they only appeared in mammals from exogenous sources in the diet. In the early 1980s, some data was obtained that suggested that an agent which inhibited NKA was present in the serum of experimental rat models (de Wardener and Clarkson, 1985). It took almost another decade to identify the circulating compound as ouabain or an isomer of it Hamlyn et al. (1991). However, several other endogenous steroids, collectively termed digitalis-like compounds (DLC), were also identified in human tissues. There is ample evidence now that CTS circulate in the bloodstream of mammals including ouabain, which appears to be endogenously produced in tissues, such as the adrenal glands and hypothalamus and that its secretion is regulated by different nervous system and humoral factors (Blaustein, 1996; Ferrandi et al., 1997; Manunta et al., 2001; Schoner, 2002; Manunta et al., 2005; Schoner and Scheiner-Bobis, 2007a; Hamlyn and Manunta, 2011). The discovery that ouabain itself or ouabain-like substances are present in plasma of humans and could function as hormones, along with the results indicating the signaling role of NKA, sparked curiosity for the physiologic implications of this new pathway in cell regulation and its potential pharmacologic relevance. A general discussion of the actions of CTS in different tissues follows.

## NKA signaling in normal physiology

Currently, a breath of information has been gathered showing that CTS have pleiotropic effects, triggering a variety of responses in different cells and tissues. Earlier studies showed that low doses of ouabain led to the growth of myocardial cells and that this could contribute to the beneficial effect of cell hypertrophy in cardiac insufficiency (Kometiani et al., 1998; Liu et al., 2000). More recently, it was shown that ouabain also delayed and even prevented the cardiac remodeling that follows pressure overload in mouse hearts. These effects are particularly important to understand the consequences of cardiac injury and the mechanisms involved in the development of heart failure (Belliard et al., 2016; Lopatina et al., 2016; Buzaglo et al., 2019). Moreover, ouabain administered prior to sustained ischemia confers protection against cardiac ischemia-reperfusion injury (Belliard et al., 2016).

Ouabain also has effects beyond the heart, causing cell proliferation of vascular smooth muscle cells (Aydemir-Koksoy et al., 2001; Abramowitz et al., 2003; Allen et al., 2003; Kotova et al., 2006b), human endothelial cells (Tverskoi et al., 2016), rat and opossum kidney tubular epithelial cells (Dmitrieva and Doris, 2003; Khundmiri et al., 2006), and fibroblasts (Winnicka et al., 2010). Endogenous ouabain also regulates viability of a series of cell lines in culture (Dvela et al., 2012). In addition, ouabain protects renal cells from undergoing apoptosis (Dmitrieva and Doris, 2003; Golden and Martin, 2006; Khundmiri et al., 2006; Li et al., 2006; Aperia, 2012), and promotes epithelial cell migration and ciliogenesis (Larre et al., 2011; Verdejo-Torres et al., 2019). Importantly, ouabain-induced NKA signaling regulates cell-cell adhesion and influences the polarized phenotype of renal epithelial cells in culture. In MDCK cells, nanomolar concentrations of ouabain enhance tight-, adherens-, and gap-junctional proteins, promoting the estable structure of the epithelium. In contrast, in the same cells, micromolar concentrations of ouabain induces cell detachment (Larre et al., 2010; Castillo et al., 2019; Contreras et al., 2024). In LLC-PK1 renal cells, NKA signaling regulates the metabolic capacity of the cells (Kutz et al., 2021). In the whole kidney, NKA signaling activated by ouabain controls vascular tone and sodium homeostasis (Cai H. et al., 2008; Nesher et al., 2009; Blaustein and Hamlyn, 2010). Ouabain also has a protective role against the harmful effects of serum deprivation and Shiga toxin infection in rat kidney tubular cells (Li et al., 2006; Li et al., 2010).

In smooth muscle cells of vessels, ouabain induces overall contraction and potentiates vascular tone and the contractile response to agonists (Zhang et al., 2018). In those cells, ouabain also regulates intercellular communication by modulating gap junctions and connexin levels (Hangaard et al., 2017). In vascular endothelial cells, ouabain promotes endothelin-1 and nitric oxide production, which further supports the notion that ouabain is a regulator of vascular tone (Scheiner-Bobis et al., 2006).

In the gastrointestinal tract, NKA plays a primary role in nutrient and water absorption by creating the Na^+^ gradient that is used by secondary transport systems (Field, 2003; Nepal et al., 2021). Also, by secondarily maintaining intracellular Ca^2+^ concentrations, NKA affects intestinal contractility (Daniel et al., 2009). Circulating ouabain regulates intestinal tight junctions by modulating claudin expression, likely via Rho-associated coiled-coil forming kinase (ROCK) signaling (Markov et al., 2020; Cho et al., 2025). Ouabain also reduces blood flow and oxygen consumption in the intestine by acting on blood vessels (Pawlik et al., 1974). Moreover, chronic exposure to ouabain appears to have a protective effect on the epithelial gut barrier (Markov et al., 2023).

Within the nervous system, ouabain increases the growth and survivability of retinal cells, neurons, and astrocytes and stimulates neuronal branching in hippocampal cells (Murata et al., 1996; Dvela et al., 2012; Li and Zhou, 2015; Lopachev et al., 2016; Salles von-Held-Ventura et al., 2016; Orellana et al., 2018).

In the lung, ouabain is involved in airway remodeling and facilitates injury by inducing proinflammatory cytokine production (Silva et al., 2023).

NKA signaling also seems to play a role in the regulation of the immune system, as CTS can affect the function and proliferation of lymphocytes and monocytes (Hammarstrom and Smith, 1979; Brodie et al., 1995; Olej et al., 1998; Esteves et al., 2005; Rodrigues-Mascarenhas et al., 2009; Kinoshita et al., 2014; Nascimento et al., 2014; Teixeira and Rumjanek, 2014). Ouabain also stimulates the secretion of interleukin in the brain (Pirkmajer et al., 2020).

Within skeletal muscle, ouabain works synergistically with insulin to enhance glycogen synthesis (Kotova et al., 2006a) and prevents injury-associated cell depolarization (Kravtsova et al., 2020; Kravtsova et al., 2021).

Ouabain participates in promoting the differentiation of different cell types, including bone marrow cells, murine stem cells (Lee et al., 2011; Sayed et al., 2014). In addition, ouabain has different effects in male and female reproductive tissues, placenta and the embryo (Thundathil et al., 2006; Uddin et al., 2008; Giannatselis et al., 2011; Lucas et al., 2012; Dvela-Levitt et al., 2015; Upmanyu et al., 2016; Upmanyu et al., 2019).

Besides ouabain, the CTS marinobufagenin has been shown to activate collagen-1 production in heart fibroblasts, stimulate epithelial to mesenchymal transition of renal epithelial cells, and promote the growth of liver tissue explants (Fedorova et al., 2009; Penniyaynen et al., 2015).

These examples show the wide variety of actions that ouabain and other CTS exert. Since most of these effects are related to stimulation of cell growth, one could consider endogenous CTS as growth hormones. However, CTS effects go beyond its cell hyperplastic and hypertrophic effects and influence other important aspects of cell physiology, including cell metabolism, migration, differentiation, apoptosis, and tissue fibrosis. A brief summary of some of the CTS actions is presented in Table 1.

**TABLE 1:** NKA signaling effects and intracellular pathways activated by CTS in different normal cells and organs.

Cell/tissue type	CTS used	Effect	Pathway involved	Reference
Cardiac myocytes	Ouabain	Hypertrophy	Src-MAPK-ERK, ROS	Xie (2001), Liu et al. (2007a)
Heart	Ouabain	Increase in muscle contractility protection against ischemia‐reperfusion injury	Src-MAPK-ERK, ROS	Buzaglo et al. (2019), Belliard et al. (2016)
Vascular smooth muscle cells	Ouabain	Cell growth Contractility High blood pressure	Src, EGFR, phosphatidylinositol-4,5-bisphosphate 3 kinase (PIP3), IP3R, Calcium, ROS	Zhang et al. (2018), Zhang et al. (2019), Pulina et al. (2010), Allen et al. (2003), Blaustein and Hamlyn (2010)
Vascular endothelial cells	Ouabain	Anti-apoptotic Stimulation of endothelin-1 and nitric oxide production	Akt	Scheiner-Bobis et al. (2006)
Renal tubular epithelial cells	Ouabain	Cell growth, regulation of cell adhesion and cell migration Protection against Shiga toxin	Src, EGFR, PIP3 kinase, IP3R, Calcium	Larre et al. (2010), Castillo et al. (2019), Dmitrieva and Doris. (2003), Verdejo-Torres et al. (2019), Burlaka et al. (2013)
Kidney	Ouabain, MBG	Regulation of vascular tone and sodium homeostasis, protection from apoptosis Renal fibrosis Protection from malnutrition	Calcium, NF-κB Src	Blaustein and Hamlyn (2010), Fedorova et al. (2009), Li et al. (2010)
Brain	Ouabain	Anti-inflammatory effect in the rat hippocampus Dendritic branching and memory	NF-κB signaling	Kinoshita et al. (2014), Orellana et al. (2018)
Neuronal cells	Ouabain	Growth Protection against apoptosis Neuronal maturation Cell viability	Bcl-2 MAP-kinase ERK1/2	Golden and Martin (2006), Lopachev et al. (2016), Dvela et al. (2012)
Astrocytes	Ouabain	Growth	ND	Murata et al. (1996)
Retinal cells	Ouabain	Growth and survival	TNF-α and IL-1β PKC	Salles von-Held-Ventura et al. (2016), Golden and Martin (2006)
Skeletal muscle	Ouabain	Stimulates glycogen synthesis Interleukin-6 secretion	Src, ERK1/2, GSK3, Akt-mTOR	Pirkmajer et al. (2020), Kotova et al. (2006a)
Lung	Ouabain	Regulation of inflammation and lung injury	MAPK, ERK, PI3K, NF-κB	Silva et al. (2023), Galvão et al. (2022)
Salivary gland	Ouabain	Potentiation of cholinergic effect	ERK1/2, Src, PKC, calcium	Plourde et al. (2006)
Liver tissue explants	MBG	Cell proliferation	ND	Penniyaynen et al. (2015)
Lymphocytes and monocytes	Ouabain Lanatoside-C	Regulation of proliferation and cytokine production	ND	Rodrigues-Mascarenhas et al. (2009), Hammarstrom and Smith (1979)
Bone marrow cells	MBG	Regulation of cell differentiation	ERK	Sayed et al. (2014)
Skin fibroblasts	digoxin, ouabain, MBG	Increases in collagen synthesis	Src	El-Okdi et al. (2008)
Murine stem cells	Ouabain	Differentiation	ERK1/2	Lee et al. (2011)
Sertoli cells	Ouabain	Cell proliferation	ERK1/2, MAPK, PIP3-kinase, Akt	Lucas et al. (2012)
Leydig cells	Ouabain	Stimulation of steroidogenesis	ERK1/2, cAMP response element-binding protein (CREB), Activating Transcription Factor 1 (ATF-1)	Upmanyu et al. (2019)
Male germ cells	Ouabain	Increased expression of integrins	ERK1/2, CREB, ATF-1	Upmanyu et al. (2016)
Spermatozoa	Ouabain	Inhibition of motility, protein phosphorylation	Tyrosine kinase	Thundathil et al. (2006)
Blastocyst	Ouabain	Regulation of tight junctions in trophoectoderm	Src	Giannatselis et al. (2011)
Placenta	MBG	Inhibition of proliferation, migration and invasion of cytotrophoblast cells in culture	ERK1/2 (downregulation)	Uddin et al. (2008)
Embryo	Ouabain	Growth and development	ERK1/2, p-90RSK, Akt, PCNA, and Ki-67	Dvela-Levitt et al. (2015)
Enterocytes	Ouabain	Cell proliferation, regulation of tight junction	PGE2, PI3K, PKC, PKA, NF-κB, MAPK, TNF-α	Nepal et al. (2021), Zeineddine and Kreydiyyeh, (2024), Serhan and Kreydiyyeh, (2011)

It is important to note that the effects of CTS are seen at concentrations that are within those at which these compounds circulate in the bloodstream of mammals (Hamlyn, 2014; Blaustein and Hamlyn, 2024). This highlights the physiologic relevance of NKA signaling for cells and tissues. The dose-dependent effects of CTS, with high relatively concentrations being deleterious and relatively low amounts inducing signaling show the functional duality of NKA, as an ion transporter essential for maintaining life and as a signaling molecule that regulates cell physiology (Blaustein, 2018). This versatility of NKA function, also shows the complex mechanisms by which NKA controls cell physiology. Below, we discuss some of the cellular pathways which have been identified as downstream effectors of CTS binding to NKA.

## Downstream effectors of the NKA signaling pathway

### Src/EGFR/Ras/Raf/MEK/ERK pathway

The first intracellular messengers noted to be involved in the hypertrophic effects elicited by ouabain in myocardiocytes corresponded to a common signal transduction cascade that included Ras and the mitogen-activated kinase/extracellularly regulated kinase 1/2 (MAPK/ERK1/2) (Kometiani et al., 1998). Further work expanded this pathway to include the upstream activation of the protooncogene Src kinase and the phosphorylation of the epidermal growth factor receptor (EGFR) (Haas et al., 2000; Pratt et al., 2018). Of particular interest was the finding that ouabain stimulated these pathways not just in rat cardiomyocytes but also in A7r5, L929, and importantly, HeLa cell lines at doses consistent with the relatively higher affinity for ouabain exhibited by human NKA (Askari, 2019). This suggested that NKA signaling was not only a phenomenon of rodent cells but was shared by human cell models. Xie and Askari additionally showed that the ouabain-induced effects occurred independently of changes in Na^+^ and Ca^2+^ concentrations and were mimicked when cells were incubated in medium without K^+^, which highly reduces the transport function of NKA (Liu et al., 2000), suggesting that these changes were due to pathways outside of ion transport inhibition. At the time, this newly identified intracellular signaling pathway, named the NKA signalosome, represented a breakthrough for our understanding for the mechanisms of action of CTS.

In studying the NKA/Src signaling cascade, important focus was placed on the mechanisms by which NKA activates Src kinase. It was reported that NKA and Src establish a direct interaction through two molecular domains. These include the Src SH2 domain, which binds to the CD2 region of NKA α1 and the Src kinase domain, which binds to the NKA α1 CD3 motif (Tian et al., 2006; Li et al., 2009; Ye et al., 2011; Yu et al., 2018). In this model, while NKA is in the E1 conformational state, Src remains bound to NKA through both of its interacting sites, which keeps it in an inactive status. Upon CTS binding, the stabilization of NKA in the E2 conformation frees the kinase domain of Src, which allows Src autophosphorylation and activation (Banerjee et al., 2015; Yu et al., 2018). The activated Src then phosphorylates EGFR and stimulates downstream effectors that lead to regulation of gene transcription. While there is evidence that NKA and Src colocalize and interact with one another (Liu et al., 2003; Wang et al., 2004; Kotova et al., 2006b; Liang et al., 2006; Tian et al., 2006; Nie et al., 2020), this interaction has not been found in all experimental models (Weigand et al., 2012; Clifford and Kaplan, 2013; Yosef et al., 2016). In this latter context, NKA and Src may be indirectly linked and it has been proposed that this could depend on other intermediary proteins or though changes in the ATP/ADP ratio putatively connected to NKA ion pumping in the cell (Weigand et al., 2012; Gable et al., 2014). Based on the hypothesis of a direct association between NKA and Src, a small peptide, pNaKtide, was developed (Riegel et al., 2009). This peptide contains the 20 amino acid sequence of the CD3 N-terminal domain of NKA α1 that was described to bind to Src and has been shown to block the ouabain-induced activation of Src, ERK, and hypertrophy in cardiac myocytes, but not the activation of the kinase by other ligands, such as insulin-like growth factor 1 (IGF-1). Since its initial synthesis, pNaKtide has been used in a number of studies where it has been reported to dampen the activation of the NKA/Src signaling complex in different cell and animal models (Liu et al., 2016; Hangaard et al., 2017; Drummond et al., 2018; Li et al., 2018; Cheng et al., 2019; Khalaf et al., 2019; Sodhi et al., 2023; Zong et al., 2023). Further investigation is needed to ascertain whether NKA-Src interaction can be explained by a direct or indirect model.

### Caveolae in NKA signaling

Following the discovery of the involvement of Src, EGFR, and ERK in ouabain-induced and NKA-mediated signaling, a specific caveolin-binding motif in the sequence of NKA α1 was identified. This suggested that NKA could be interacting with its signaling partners within the caveolar microdomains of the plasma membrane (Xie and Askari, 2002). Caveolae, flask-shaped invaginations of the cell membrane rich in cholesterol, are centers that harbor a series of ligand receptors and related signaling molecules (Cohen et al., 2004; Patel et al., 2008; Lamaze et al., 2017; Parton, 2018). Therefore, it was theorized that NKA may be sharing the microenvironment of caveolae with other receptors as clusters for cell signaling (Fielding, 2001; Parton et al., 2018). This was supported by the use of cholesterol-depleting agents, which showed that caveolar integrity was necessary for the downstream actions of ouabain in cardiac myocytes, including NKA/Src colocalization and activation of ERK (Liu et al., 2003; Wang et al., 2004). Additionally, it was shown that ouabain-induced endocytosis of NKA alongside Src and EGFR occurred via clathrin-coated vesicles in a manner requiring caveolae. This ouabain-dependent NKA endocytosis agrees with the common mechanism of receptor signaling inactivation (Liu, 2005; Liu et al., 2005). Moreover, knock out of caveolin-1, an essential structural component of caveolae, interfered with the ouabain-induced activation of Src, ERK, collagen synthesis, and proliferation, but did not affect Src and ERK basal activation (Quintas et al., 2010). Further, cardiac cells obtained from caveolin-1 knockout mice exhibited reduced ouabain-induced activation of PI3K/Akt, ERK, and cardiac hypertrophy, validating the idea of the need of caveolae for NKA signaling (Bai et al., 2016).

Based on the importance of caveolae in NKA signaling, it was proposed that two separate pools of NKA existed at the cell plasma membrane: one corresponding to the signaling NKA localized in caveolae and the other representing the “pumping” NKA, which performed the classical Na^+^/K^+^ exchange at the general plasma membrane (Liang et al., 2007). The relationship between NKA and caveolae was further supported by the reduction of caveolin-1 expression at the cell plasma membrane and its shift to intracellular stores when NKA expression was reduced (Cai T. et al., 2008). Additional evidence for a NKA-caveolae connection was suggested by the increase in NKA activity observed in models where cell caveolae were disrupted, which presumably indicated that the NKA free from caveolae is then dedicated to ion transport (Quintas et al., 2010). Conversely, graded reduction of NKA expression in pig kidney LLC-PK1 cells resulted in the loss of the capacity of the cells to respond to ouabain-induced signaling in favor of the more vital ion transport function of NKA (Liang et al., 2007). However, a clear difference between the signaling and pumping actions of NKA has not been supported by other reports, which have shown that NKA from both caveolar and non-caveolar preparations retain ion transport activity and reside with signaling partners (Liu et al., 2011); therefore, it remains unclear whether caveolae are strictly the place from which the NKA signaling apparatus operates or if the caveolar NKA is also involved in pumping ions.

Interestingly, isolated caveolar preparations showed that NKA α1 has a higher affinity for ouabain than preparations of general cell plasma membrane, where NKA α1 typically shows a lower capacity for ouabain binding. These findings led to the provoking idea that increased ouabain affinity of the caveolar NKA would facilitate the binding and downstream effects of ouabain (Dmitrieva and Doris, 2003; Ferrandi et al., 2004). However, another report failed to show caveolar-specific changes in the kinetics of NKA toward ouabain (Liu et al., 2011). Notably, the change in ouabain affinity was seen in caveolar fractions obtained from detergent-free methods, while the contrasting study used sodium-dodecyl-sulfate (SDS) to enrich for NKA following the classical protocol developed by Jørgensen (Jørgensen, 1986). It is possible that within the environment of cell caveolae, NKA has an increased capacity to bind CTS, but this may be disrupted by harsh methods of purification with detergents. In summary, the NKA signaling apparatus requires the association of several proteins including caveolin and the caveolae microdomains of the plasma membrane where other receptor kinases are also found (Lamaze et al., 2017).

### Reactive oxygen species (ROS)

Reactive oxygen species (ROS) were identified as other intermediates of the CTS/NKA signaling pathway of cardiac cells. It was shown that the antioxidant n-acetylcysteine (NAC) inhibited the transcriptional and translational changes of growth related genes normally increased by ouabain in cardiac myocytes, including skeletal muscle actin (skACT) and atrial natriuretic factor (ANF) (Chen et al., 2017). Notably, in these cells NAC also partially ablated the increase in ERK phosphorylation induced by ouabain, suggesting some overlap for the signaling pathways downstream of CTS binding to NKA (Aizman et al., 2001). The link between NKA signaling and ROS production was also established in human pluripotent stem cells induced to develop as myocardiocytes and expressing a mutant form of the NKA α1 that cannot signal through Src. Those cells showed significant reduction in ROS but also had reduced basal and maximal rates of mitochondrial respiration, spare respiratory capacity, and ATP production, indicating the importance of ROS as part of NKA signaling and the role of this pathway in cell metabolism (Cai et al., 2023). In the whole heart, low doses of ouabain exert cardioprotective effects and ischemia pre- and post-conditioning (Fuerstenwerth, 2014; Duan et al., 2018; Marck and Pierre, 2018). It has recently been proposed that this is mediated by NKA signaling and opening of the mitochondrial ATP sensitive channel (mitoK_ATP_), which causes the increase in ROS and secondary stimulation of the mitochondrial protein kinase C epsilon. This in turn inhibits the opening of the mitochondrial permeability transition pore (mPTP), protecting the tissue from cell death. Interestingly, it has been proposed that the connection between NKA signaling and mitochondria occurs via vesicular signaling complexes; however, this is currently a hypothesis that remains to be confirmed (Garlid et al., 2009).

ROS generation via NKA signaling activated by ouabain was found to occur in various other cell types (rat cardiomyocytes, A7r5, and HeLa cells) and to be independent from changes in intracellular Na^+^ and Ca^2+^ concentrations, further supporting the notion that NKA signaling is independent from the ion transport function of NKA (Liu et al., 2000). ROS appears to function upstream of MEK and ERK as NAC partially ablated the increases in ERK phosphorylation (Aizman et al., 2001; Yan et al., 2013).

Additionally, it was shown that NAC pretreatment of LLC-PK1 cells ablated the normal ouabain-induced increase of phosphorylation of Src, suggesting that ROS may play an essential role in NKA signaling in other tissues, such as the kidney (Yan et al., 2013). Interestingly, ROS seem to activate NKA signaling in the absence of CTS or at least act on similar pathways (Liu et al., 2006; Liu et al., 2012; Wang et al., 2014). This activation of NKA signaling by ROS results in a positive feedback mechanism, known as the ROS amplification loop, which leads to oxidative stress. This interplay between NKA signaling and ROS has been linked to worsening of chronic conditions, such as obesity, diabetes, dyslipidemia, and atherosclerosis (Srikanthan et al., 2016). This effect of ROS may be associated with redox modifications of NKA and Src. It has been shown that Src kinase can be oxidized and also glutathionylated depending on redox state and this affects Src activity (Yang et al., 2020). NKA also undergoes redox-dependent modifications through glutathionylation of its α and β subunits (Figtree et al., 2009; Petrushanko et al., 2012). Interestingly, according to modeling data, the glutathionylation of the NKA α subunit can affect the interaction of Src kinase and Na,K-ATPase (Petrushanko et al., 2017).

### Calcium oscillations and protein kinase C (PKC)

An important additional intermediate of NKA signaling was identified by the group of Aperia in kidney epithelial cells. These researchers found that partially inhibitory concentrations of ouabain in rat kidney tubular cells induced regular, low-frequency calcium oscillations that caused the activation and nuclear localization of the transcription factor NF-κB (nuclear factor kappa-light-chain-enhancer of activated B cells) (Aizman et al., 2001). Their study also supported the involvement of phospholipase C as part of the (PLC)/inositol triphosphate receptor (IP3R) pathway. PLC had also been implicated in the effects of ouabain in rat cardiac myocytes (Kometiani et al., 1998). Studies using preparations from rat kidney confirmed that PLC-1 and the IP3R isoforms 2 and 3 were coenriched with NKA, caveolin-1 and Src in light density fractions of the cell membranes. It was also found that the major intracellular domain of NKA interacts with PLC-γ1, while the N-terminus binds to IP3R, which suggested that NKA may tether PLC-γ1 and IP3 receptors to form a calcium regulatory complex (Zhang et al., 2006). NKA and IP3R interaction is stabilized by ankyrin (Liu et al., 2008; Aperia et al., 2020) and this is enhanced by the presence of ouabain (Miyakawa-Naito et al., 2003). Importantly, ouabain stimulated the phosphorylation of PLC-γ1 and IP3 in a Src dependent manner and the release of Ca^2+^ from intracellular stores in LLC-PK1 cells (Yuan et al., 2005). The direct association between NKA and IP3R also suggested that NKA served to bring together the ER and cell plasma membrane to facilitate calcium signaling to discrete regions of the cell (Chen et al., 2008).

The involvement of calcium as a messenger in the NKA signaling pathway was also found in neurons. Thus, ouabain enhances dendritic growth of rat cortical brain cells via calcium oscillations, MAP kinase, Ca-calmodulin dependent protein kinase, and activation of gene transcription regulated by cAMP response element binding protein (CREB) (Desfrere et al., 2009). Ouabain also generates calcium oscillations in glial cells, activating the IP3R and NF-κB in hippocampal astrocytes in culture (Liu X. L. et al., 2007). In conclusion, the experimental evidence available indicates that ouabain-induced and NKA-mediated signaling activates IP3R in two ways: one through stimulation of PLC, and the other by a direct interaction of NKA with IP3R. It is important to note that that the effects of CTS involve NKA control of intracellular calcium levels through both inhibition of NKA ion transport activity and signaling. These mechanisms cooperate in fine tuning intracellular calcium by decreasing its export out of the cell and favoring its movement from intracellular stores (Tian and Xie, 2008). Another point to consider is that calcium signaling results from a complex interplay between activation and inactivation of intracellular and cell surface calcium permeable channels, each subjected to specific regulation (Dupont et al., 2011; Parkash and Asotra, 2012; Smedler and Uhlen, 2014). Therefore, CTS represent additional regulators, which along with others control the development and amplitude of calcium spikes, as well as the frequency of calcium oscillations. It is interesting to propose that the low frequency calcium changes induced by ouabain represent a signature of the action of CTS that leads to cell effects that are different from those caused by other stimuli.

### PI3K/Akt/mTOR pathway

The effects of ouabain on cell hypertrophy and proliferation suggested that protein kinase B (Akt), a mediator known to influence cell growth, could be involved in NKA signal transduction. Working with opossum kidney proximal tubular cells, it was found that nanomolar concentrations of ouabain stimulated Akt in a manner that required phosphatidylinositol 3 kinase (PI3K), PLC, and Ca^2+^ (Khundmiri et al., 2006). These authors also showed that Akt inhibition suppressed the ouabain-induced increase in cell proliferation. Shortly after, experiments performed in neonatal and adult rat cardiac myocytes, as well as in adult rat Langendorff-perfused hearts, showed that ouabain stimulated the phosphorylation of Akt and its substrates, mammalian target of rapamycin (mTOR) and glycogen synthase kinase (GSK) in a manner that required PI3K 1A (Liu L. et al., 2007). It was also found that this pathway required Src but not EGFR or MEK, suggesting that the activation of Src by ouabain stimulates PI3K and Akt signaling independently from EGFR and ERK. However, a later study using SYF cells, a line deficient for the kinases Src, Yes, and Fyn, and the same cells where Src has been reintroduced, showed that ouabain-induced activation of Akt was independent from the presence of Src (Wu et al., 2013). This study also provided evidence for a direct interaction between NKA and PI3K 1A. This indicated that NKA signaling uses downstream pathways that are separate from the activation of Src.

## Regulation of NKA signaling

In the short term, ouabain and other CTS at circulating concentrations do not cause a measurable change in NKA pumping activity; however, it has been noticed that chronic treatment with CTS decrease NKA pumping activity and, in the kidney, it reduces Na^+^ absorption; interestingly, this occurs without significant changes in intracellular Na^+^ (Liu et al., 2002; Liu, 2005). The ouabain-induced decrease in NKA activity was shown to be due to endocytosis of NKA via a clathrin-coated pit and endosome-dependent process, which requires Src, PI3K, and caveolin-1 (Liu et al., 2004; Liu et al., 2005). Interestingly, not only NKA but also other components of the NKA signaling complex are internalized, as suggested by the finding of EGFR and Src, along with NKA, in endosomes (Liu, 2005). Once brought into the cell, the fate of the NKA signaling complex is its degradation in lysosomes. This process is of physiological relevance, since it serves to regulate NKA signaling to avoid overstimulation of the NKA signaling pathway (Cherniavsky-Lev et al., 2014). At present, the mechanisms that lead to NKA internalization are unclear. It has been suggested that this process may be due to a conformational change in NKA itself, caused by the binding of CTS, rather than activation of additional intracellular signaling cascades. This is supported by evidence showing that several stimuli other that CTS, such as hypoxia and hypercapnia, also cause NKA internalization (Chibalin et al., 1997; Zhang et al., 1999; Dada et al., 2003; Lecuona et al., 2009; Welch et al., 2010). It is possible that the conformational state of NKA depends on the type of CTS bound. In particular, ouabain and marinobufagenin binding drives NKA into different conformations, which could help explain the diversity of cellular effects that different CTS have (Klimanova et al., 2015). Thus, ouabain and marinobufagenin, at similar concentration, equally inhibit NKA ion transport in renal epithelial cells; however, they induce cell death at different IC_50_ values (Akimova et al., 2005). While further investigation is needed, it is clear that NKA internalization and degradation represents a mechanism important to not only control NKA signaling but also influence overall NKA expression levels and activity at the cell plasma membrane. This is another indication of the complex crosstalk that exists between CTS-induced NKA signaling events and NKA pumping activity.

A factor that modulates NKA signaling is hypoxia. It has been shown that under low oxygen conditions, the activation of mediators of NKA signaling, such as Src and ERK is abrogated in human embryonic kidney cells (HEK293 cells) and mouse fibroblast cells (SC1 cells) (Lakunina et al., 2017; Petrushanko et al., 2017). Therefore, hypoxia makes the cells less sensitive to CTS, reducing the effects of these substances. Conversely, ouabain protects SC1 cells from hypoxia (Lakunina et al., 2017) and this effect has also been observed in neuronal slice cultures (Oselkin et al., 2010). Endogenous CTS play an important role in adaptation to hypoxia and their level increases in human divers and diving animals (Manfrini et al., 2022). Under conditions of chronic hypoxia, such as in patients with idiopathic pulmonary arterial hypertension, endogenous CTS are increased (Manfrini et al., 2023). Moreover, endogenous CTS are also elevated in myocardial and renal ischemia (Strauss et al., 2019). Altogether, this shows that ouabain prevents the damage caused by low oxygen and ischemia and therefore, CTS may provide a beneficial effect in the adaptation of cells to hypoxia.

## Signaling of NKA α isoforms

Most studies of the signaling properties of NKA have focused on the NKA α1, which is the NKA α isoform expressed in most cell types (Blanco and Mercer, 1998). Initial studies focused on the use of relatively high concentrations of ouabain; however, as studies on NKA signaling developed, lower doses of ouabain were used, which better reflected the CTS levels normally found in the bloodstream of mammals (in the picomolar to nanomolar range). The relatively low affinity of NKA α1 for ouabain in rodent models posed the controversy as to how this NKA isoform could respond to the low amounts of ligand used in some experiments. As will be discussed below, this was explained by the requirement of only a few molecules of ouabain to bind to NKA and that this will result in an amplification through the activation of the downstream mediators. However, this hypothesis has not yet been unequivocally demonstrated. Treatment of adult or neonatal cardiac myocytes with ouabain, in doses lower that those used in initial experiments, recapitulated the observations originally obtained. Since in addition to NKA α1, myocytes also express NKA α2 and NKA α3 isoforms (Blanco and Mercer, 1998), focus was directed to the role of NKA α isoforms different from NKA α1. This was logical, considering that the higher affinity of NKA α2 and NKA α3 would allow these isoforms to more readily respond to lower concentrations of ouabain. However, it was shown that NKA signaling in cardiac cells was primarily mediated via NKA α1, while the changes in intracellular Ca^2+^ due to ouabain ion transport inhibition of NKA α2 was responsible for the inotropic effects of CTS (Blaustein and Hamlyn, 2020). Additional studies performed in Sf9 insect cells expressing NKA α2 suggested that this NKA isoform was not involved in signaling. In addition, in this cell model, NKA α1, NKA α3, and NKA α4 responded to ouabain by increasing the phosphorylation of ERK in a dose-dependent manner and in line with the respective affinity that each NKA isoform has for ouabain (Pierre et al., 2008). In agreement with the findings in Sf9 cells, LLC-PK1 cells with severe knock down of NKA α1, and rescued with rat NKA α2, were able to recover ion transport function but failed to reestablish ouabain-stimulated signaling (Xie et al., 2015). This suggested that the inability of NKA α2 to signal was not an artifact produced in the insect cells but also occurs in mammalian cells. Different from these results, in smooth muscle cells of the vasculature, NKA α2 responds to ouabain not only reducing ion transport, but also by interacting with Src and activating downstream signal transduction pathways (Kotova et al., 2006b; Bouzinova et al., 2018). Therefore, it appears that the ability of NKA α2 to signal is tissue and cell type specific.

An interesting consideration when analyzing the involvement of NKA α2 in signaling CTS effects is that the putative Src interaction domain present in NKA α1 is absent in NKA α2 (Xie et al., 2015). Interestingly, when NKA α2 was mutated to express the Src interacting domain of NKA α1 in kidney epithelial cells, ouabain was then able to activate ERK in the cells (Yu et al., 2018). It is therefore possible that NKA α2 does have signaling capacity, but it uses downstream effectors that are different from those activated by the other NKA isoforms.

Unlike NKA α2, NKA α3 has been found to increase ERK phosphorylation in response to sub-inhibitory concentrations of ouabain in systems where NKA α3 is either the only or the predominant α isoform expressed (Pierre et al., 2008; Madan et al., 2017). In the LLC-PK1 model, in which pig NKA α1 is greatly knocked down, expression of rat NKA α3, reestablishes ouabain induced phosphorylation of ERK and Akt. Interestingly, this response did not involve Src and instead relied on the activation of PKC and PI3K. In this manner, unlike NKA α1, NKA α3 signaling is not Src-based (Madan et al., 2017). Studies in neuroblastoma cells, which contain both NKA α1 and NKA α3, have demonstrated an increase of ERK activation with ouabain administration. When these two isoforms were individually silenced with small interfering RNA (siRNA), ERK activation was abolished only when NKA α3 but not NKA α1 was silenced. (Karpova L. et al., 2010). In another study, treatment of rat cerebellar cells with relatively low ouabain concentrations led to activation of the MAPK, PKC and PIP3 kinases, causing cell apoptosis. Instead, higher concentrations of ouabain stimulated Src and MAPK and reduced apoptosis. Based on the differences in ouabain affinity for NKA isoforms, it was interpreted that that NKA α1 and NKA α3 mediate ouabain effects using different pathways (Karpova L. V. et al., 2010). Differences in NKA α3 and NKA α1 signaling were also suggested by the transcriptomic changes triggered by ouabain in rat cerebellar granule cells (Smolyaninova et al., 2019). Altogether, these results show the selective role that different NKA isoforms exert in mediating ouabain effects. The fact that NKA α3, similar to NKA α2, lacks the putative Src-binding domain present in NKA α1 supports the idea that NKA α3 uses other downstream effector and that this may be occurring in a cell type specific manner (Madan et al., 2017).

Finally, the signaling capacity of NKA α4, an isoform which is only expressed in male germ cells of the testis and primarily in differentiated sperm, has also been explored. Exposure of murine spermatogenic cells (GC-2) to ouabain increased ERK phosphorylation, the transcription factors CREB, ATF-1, and the expression of integrins in the cells. This response was abolished when NKA α4 expression in those cells was reduced via siRNA (Upmanyu et al., 2016). Studies performed in differentiated spermatozoa from bull showed that ouabain stimulated the phosphorylation of a series of proteins at tyrosine residues and aided in the process of sperm capacitation, an event that is important for sperm fertilizing capacity (Thundathil et al., 2006; Rajamanickam et al., 2017). However, these authors used concentrations of ouabain which are significantly higher than the ouabain concentrations detected in body fluids, which makes uncertain whether this is a physiological regulatory mechanism for sperm. Because bull spermatozoa contain the NKA α1, NKA α2, and NKA α3 isoforms in addition to NKA α4, dissecting the contribution of each NKA isoform to ouabain’s effects is difficult. However, NKA α4 signaling capacity is supported by its interaction with caveolin-1 and EGFR in the lipid raft fractions, and with SRC, EGFR, and ERK in the non-raft fractions isolated from the membranes of those cells (Rajamanickam et al., 2017).

**FIGURE 2:**
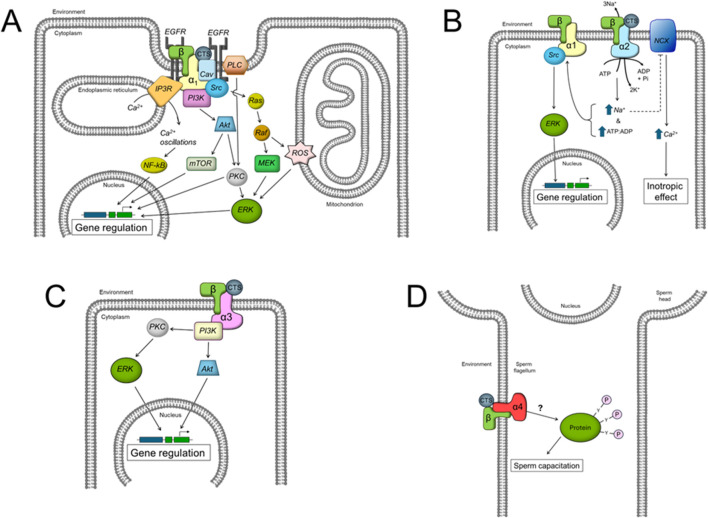
Scheme showing the different intracellular signaling pathways described to date for the effects of CTS by the different NKA α isoforms. **(A)** α1 isoform, **(B)** α2 isoform, **(C)** α3 isoform, and **(D)** α4 isoform. See the text for additional definitions.

Altogether these results suggests that each NKA α isoform uses different downstream effectors to influence cell function in a specific manner. The specific signaling pathways known to date for each NKA α isoform are illustrated in Figure 2.

## NKA signaling in disease

As NKA signaling was further explored, focus was placed not only on the effects of CTS in normal states, but also under different pathological situations. Below, we summarize conditions in which CTS induced and NKA mediated signaling is altered, either due to altered levels of circulating CTS or to abnormal response of the diseased cells (see Table 2).

**TABLE 2:** NKA signaling effects and intracellular pathways activated by CTS in disease conditions.

Disease	Cell/tissue type	CTS used	Effect	Pathway involved	Reference
Chronic Kidney Disease	Kidney	MBG, TCB, Ouabain	Reduced kidney mass, high blood pressure, fibrosis, inflammation	Src, NF-κB, ROS	Graves et al. (1983), Kennedy et al. (2008), Komiyama et al. (2005), Kolmakova et al. (2011), Kennedy et al. (2018), Khalaf et al. (2019), Khalaf et al. (2020), Wang et al. (2019), Fedorova et al. (2009), Haller et al. (2014), Elkareh et al. (2007), Kennedy et al. (2006), Bolignano et al. (2022), Maxwell et al. (2021)
Autosomal Dominant Polycystic Kidney Disease	Kidney	Ouabain	Fluid filled cyst formation in kidney and other organs	Src, ERK, PI3K, NF-κB, EGFR	Nguyen et al. (2007), Trant et al. (2023), Nguyen et al. (2011), Jansson et al. (2012), Jansson et al. (2015), Venugopal and Blanco (2016), Venugopal et al. (2017), Jansson et al. (2013), Trant et al. (2025)
Hypertension	Cardiovascular System	Ouabain	High blood pressure, blood vessel strain, elevated risk of heart attack	Src, calcium	Manunta et al. (2001), Manunta et al. (2005), Hamlyn et al. (1982), Yamada et al. (1994), Ferrandi et al. (1992), Huang and Leenen (1998), Fedorova et al. (2010), Ferrandi et al. (2004), Kaide et al. (1999), Krep et al. (1995), Blaustein et al. (1998), Bagrov et al. (1995), Fedorova and Bagrov (1997), Bagrov and Fedorova (1998), Bagrov and Fedorova (2005), Blaustein and Hamlyn (2010), Bouzinova et al. (2018), Hangaard et al. (2017), Zhang et al. (2018), Zhang et al. (2019)
Pre-eclampsia	Placenta	Digitalis, MBG, Ouabain	Hypertension, fibrosis, increased flood retention	Fli-1	Mottelson et al. (2020), Graves (1987), Lopatin et al. (1999), Averina et al. (2006), Reznik et al. (2019), Morris et al. (1988), Amler et al. (1994), Di Grande et al. (1993), Goodlin (1988), Adair et al. (2010), Adair et al. (2009), Fedorova et al. (2018)
Retinal Dystrophy	Retina	Ouabain	Hereditary form of macular degeneration, loss of vision	Src, ERK, PLC, IP3R, SARM1	Molday et al. (2007), Friedrich et al. (2011), Plössl et al. (2017a), Plössl et al. (2017b), Schmid et al. (2020)
Bipolar Disorder	Brain	Digitalis, Ouabain	Abnormally elevated mood, manic episodes, depression	ERK, MEK, Akt	Goldstein et al. (2006), Grider et al. (1999), El-Mallakh et al. (2010), Keller and Frishman (2003), Logan and McClung (2016), El-Mallakh et al. (2003), Riegel et al. (2009), Sui et al. (2013), Brocardo et al. (2010), Varela et al. (2015), Goldstein et al. (2012), Hodes et al. (2016), Hodes et al. (2018), Yu et al. (2010), Kim et al. (2013), Kim et al. (2008), Lichtstein et al. (2018), Tochigi et al. (2008), Kirshenbaum et al. (2015)

### Chronic kidney disease

In the 1980s, it was noted that patients with renal failure or chronic kidney disease (CKD) had elevated circulating levels of a digoxin-like substances (Graves et al., 1983). This finding remained unexplained for a number of years, until the discovery of the function of NKA as a signaling system. By performing partial nephrectomy in mice, as a model for uremic cardiomyopathy, it was found that the reduction in kidney function was accompanied by an increase in blood pressure (Kennedy et al., 2008). Later, it was reported that patients with CKD, as well as animal models of renal failure, had elevated levels of MBG and another CTS, telocinobufagenin (TCB) (Komiyama et al., 2005; Kolmakova et al., 2011). Further investigation indicated that these substances contributed to the kidney damage that accompanies renal failure in CKD, with an increase in fibrosis as the central cause. This was demonstrated in epithelial renal cell lines and in rodent models of CKD, where fibrosis required Src and an intact NKA signaling complex (Kennedy et al., 2018). TCB also augmented inflammatory cytokines, macrophage oxidative burst, and activated NF-κB (Khalaf et al., 2019). TCB effects were prevented if mice were manipulated to reduce expression of NKA α1 or when cell lines were pretreated with the NKA/Src complex inhibitor pNaKtide or the Src inhibitor PP2. TCB also increased macrophage adhesion to cultured renal cells and upregulated inflammatory adhesion molecules in immune and endothelial cells, effects which were blocked by pNaKtide and NKA α1 knockdown (Khalaf et al., 2020). Ouabain, similar to TCB, also worsened renal damage in nephrectomized rats through activation of the Src signaling pathway, increase in oxidative stress, and stimulation of the inflammasome (Wang et al., 2019).

The role of MBG was also studied in the development of the cardiovascular damage that follows renal failure (Kolmakova et al., 2011). MBG increases the nuclear translocation of the EMT transcription factor Snail in rat hearts. This also occurred in LLC-PK1 cells, where MBG increased the expression of other EMT proteins, including collagen I, fibronectin, and vimentin (Fedorova et al., 2009). Studies in rats have shown that in renal insufficiency secondary to partial nephrectomy, treatment with a monoclonal antibody against MBG or with Digifab, a digoxin antibody and nonspecific CTS binder, reduced kidney damage (Haller et al., 2014). These results suggest that elevated levels of CTS in the setting of renal failure exacerbate renal damage, especially through inflammation and fibrosis.

MBG is also implicated in the development of uremic cardiomyopathy due to renal failure in rats. Thus, administration of low doses of MBG caused the same extent of uremic cardiomyopathy as partial nephrectomy (Elkareh et al., 2007). MBG increased blood pressure and heart size, impaired diastolic relaxation, and enhanced cardiac fibrosis. In this model, pre-immunization against MBG attenuated the cardiac hypertrophy, impairment of diastolic function, cardiac fibrosis, and systemic oxidant stress seen with partial nephrectomy without a significant effect on blood pressure (Kennedy et al., 2006). MBG also directly affected isolated cardiac fibroblasts, increasing collagen expression and procollagen stability, which was ablated by the addition of inhibitors of Src, tyrosine phosphorylation, EGFR, and ROS formation (Elkareh et al., 2007). In agreement with the data described above, a correlation between MBG and cardiomyopathy was observed in patients with end-stage kidney disease (Bolignano et al., 2022). It has been further shown that erythrocytes from CKD patients had lower NKA activity, potentially due to the reduction in NKA at the cell surface do to internalization caused by the chronically increased circulating levels of CTS (Kolmakova et al., 2011). This NKA retrieval from the cell plasma membrane is dependent on the increased production of ROS, through NKA signaling (Maxwell et al., 2021). It is possible that this phenomenon contributes to the anemic phenotype that accompanies CKD.

In conclusion, the elevation of CTS in CKD is detrimental to patients with this condition, exacerbating renal insufficiency and the associated cardiomyopathy. Progression of these conditions appears to depend on an aberrantly activated NKA signaling apparatus in the setting of CKD. Therefore, it is conceivable that treatments directed toward sequestering the excess circulating CTS or that can dampen NKA-based signaling could alleviate CKD symptoms.

### Autosomal dominant polycystic kidney disease

In the process of exploring the role of NKA in autosomal dominant polycystic kidney disease (ADPKD), our laboratory discovered that ouabain induced NKA signaling significantly contributes to the progression of this disease. ADPKD is the most common hereditary disease of the kidney, characterized by the formation and growth of fluid-filled cysts in the kidney and other organs. In human primary ADPKD cells isolated from renal cysts of patients with the disease and in renal tissue from mouse models of ADPKD, we discovered that a subpopulation of NKA has a significantly higher affinity for ouabain (Nguyen et al., 2007; Trant et al., 2023). This subset of NKA, which is constituted by the NKA α1 isoform, is characterized by an IC_50_ for ouabain that is 2-3 orders of magnitude lower than the NKA α1 from the normal kidney. Based on its response to ouabain, we estimated that this NKA population comprises between 15%–20% of the total NKA present in the sample (Nguyen et al., 2007). This higher-than-normal affinity for ouabain allows for relatively low doses of ouabain (in the nM range) to stimulate cell proliferation of human ADPKD cells in culture, but not in normal human kidney (NHK) cells (Nguyen et al., 2011). Besides increasing ADPKD cell growth, ouabain also stimulated the cyclic AMP (cAMP)-dependent increase in fluid secretion in ADPKD monolayers, another key event in the formation of ADPKD cysts (Jansson et al., 2012; Jansson et al., 2015). Interestingly, other cell parameters known to be accelerated in ADPKD, such as cell apoptosis (though to a lesser degree than proliferation) and epithelial to mesenchymal transition (EMT), are also promoted by ouabain (Venugopal and Blanco, 2016; Venugopal et al., 2017). These effects are mediated by activation of the classical NKA signaling pathway, involving Src, EGFR, MEK, and downregulation of cell cycle dependent kinases, which are already abnormally active in ADPKD (Jansson et al., 2013). In this manner, ouabain is a factor that further impacts the intracellular growth pathway that promote ADPKD cystogenesis. These data represented one of the first pieces of evidence for NKA signaling as a mechanism that, when altered, can exacerbate the progression of disease.

We have also investigated the effects of ouabain-induced NKA signaling in slowly progressive mouse models of ADPKD. We have shown that low doses of ouabain significantly increase cyst progression *in vivo* and that ouabain-induced cyst progression depends on the ouabain affinity of NKA, as cyst progression drastically increases in a mouse model where all NKA α1 has been genetically engineered to have a high affinity for ouabain (Trant et al., 2023). Recently, we have shown that these ouabain effects also require cell caveolae, and that NKA signaling in ADPKD is abrogated when caveolae are not present (Trant et al., 2025). Therefore, ouabain at levels such as those circulating in blood, and through the higher-than-normal affinity of NKA of ADPKD cells, promotes renal cyst formation and development.

### Hypertension

Increased circulating concentration of CTS have been reported both in rodent models with volume expansion and high blood pressure and in patients with essential hypertension (Hamlyn et al., 1982; Ferrandi et al., 1992; Yamada et al., 1994; Huang and Leenen, 1998; Manunta et al., 2001; Manunta et al., 2005; Fedorova et al., 2010). Moreover, intravenous infusion of ouabain into rats caused a significant increase in blood pressure (Ferrandi et al., 2004). This hypertensive state could be prevented if the animals were pre-treated with Digifab (Krep et al., 1995; Kaide et al., 1999). These observations prompted investigations to study the mechanisms by which CTS and NKA signaling affect the vasculature. The role of ion changes and the typical increase in calcium via the NKA and Na/Ca exchanger link was shown to occur in vascular smooth muscle following a mechanism similar to that of cardiac cells. Blaustein showed that NKA α2 is the main NKA α isoform involved in controlling Na^+^ and Ca^2+^ concentrations in the restricted space between the cell plasma membrane and the sarcoplasmic reticulum, which he called the plasmerosome (Blaustein et al., 1998). Bagrov found that not only ouabain but also marinobufagenin exerts a similar effect in rat aorta and that this depends on the action on NAK α1 from the smooth muscle and NAK α3 isoform from nerve terminals arriving to the vessel (Bagrov et al., 1995; Fedorova and Bagrov, 1997). Moreover, ouabain and marinobufagenin can also regulate the tone of mesenteric arteries (Bagrov and Fedorova, 1998), showing that the coordinated action of both NKA ligands controls blood pressure (Bagrov and Fedorova, 2005). This explained how circulating ouabain, by preferentially inhibiting the pumping action of the ouabain-sensitive NKA α2 causes hypertension (Blaustein and Hamlyn, 2010). However, mechanisms besides NKA pumping action are also involved. Of interest was the observation that ouabain potentiates vascular tone and the contraction of vessels induced by vasoconstrictor agonists (Bouzinova et al., 2018). This effect is mediated through NKA signaling within vascular smooth muscle cells. Ouabain increases input resistance in the arterial wall of vascular smooth muscle cells which is antagonized by inhibitors of tyrosine phosphorylation, Src, and the NKA/Src complex. It also increases Src autophosphorylation, which is antagonized by pNaKtide (Hangaard et al., 2017; Zhang et al., 2018), and sensitizes the contractile apparatus of the cells to calcium through phosphorylation of myosin phosphatase targeting protein 1 (MYPT1) (Bouzinova et al., 2018). The effect of ouabain on the potentiation to noradrenaline sensitivity correlated with arterial size and ouabain-induced Src activation (but not total Src levels). The vasoconstriction appears to be due to sensitization of the muscle contractile machinery rather than changes in intracellular calcium. In addition, arterial size also correlated with a higher number of high affinity ouabain binding sites as measured with the fluorescent marker BODIPY FL ouabain, which implicates NKA α2 over NKA α1 as the main isoform involved in this process. Considering that relatively low doses of ouabain were used in the study, which would inhibit the ion transport of NKA α2 more than NKA α1, the authors concluded that local but not general changes in Na^+^ concentration, due to NKA α2 inhibition, may be mediating the effect (Bouzinova et al., 2018; Zhang et al., 2019). Without a doubt, NKA and the regulation of both its pumping and signaling functions by endogenous as well as exogenous CTS play a major role in the control of blood pressure.

### Pre-eclampsia

There are many physiological changes that occur during pregnancy, including fluid and Na^+^ retention, which increases overall plasma volume by 50% by the end of gestation (Mottelson et al., 2020). This change prompted several lines of research to explore the role of CTS in pregnancy as regulators of vascular resistance and renal salt and fluid balance. It was shown that a moderate increase in digitalis-like compounds occurs in a healthy pregnancy and that this increase was more drastic in pregnancies that developed with hypertension (Graves, 1987). It was later shown that the levels of marinobufagenin (MBG) in plasma correlated to severity of hypertension in pre-eclamptic patients (Lopatin et al., 1999; Averina et al., 2006; Reznik et al., 2019). The involvement of CTS in pre-eclampsia was strengthened by the finding that the placenta itself produces CTS (Morris et al., 1988). In addition, NKA expression in the placenta of pre-eclamptic/hypertensive patients was found to be increased and its affinity to ouabain slightly augmented (Amler et al., 1994). Further studies showed that Digibind decreased pre-eclamptic placental arterial tone (Di Grande et al., 1993), decreased the blood pressure of hypertensive patients during pregnancy (Goodlin, 1988; Adair et al., 2010), and ameliorated postpartum pre-eclampsia (Adair et al., 2009). It is currently thought that the increase NKA signaling worsens or even causes pre-eclampsia itself due to an increase in fibrosis, as happens in cardiac, renal, and vascular tissues. Indeed, it has been shown that MBG at elevated levels in the placenta can increase placental and umbilical collagen-1 protein as a result of dampened expression of the transcription factor Fli-1, a mechanism shared with other tissues (Fedorova et al., 2018). Despite promising results in clinical trials with CTS immunoneutralization as a treatment for pre-eclampsia, little research has been done on the downstream pathway mechanisms for NKA signaling in the placenta.

### Retinal dystrophy

Interest in the involvement of NKA in a hereditary form of retinal dystrophy, X-linked juvenile retinoschisis (XLRS), was fostered after discovering that the deficient protein in the disease, retinoschisin, directly interacts with the retinal NKA α3β2 isoenzyme (Molday et al., 2007). It was later found that the interaction requires the NKA β2 subunit and that this phenomenon is important for the anchoring of retinoschisin to retinal cell membranes (Friedrich et al., 2011; Plössl et al., 2017a). When it was discovered that retinoschisin itself regulated ERK activation and its downstream target genes, interest was directed into the involvement of NKA signaling specifically in this disease (Plössl et al., 2017b). When retinoschisin was re-introduced into a model deficient in this protein, the colocalization between NKA and its signaling partners, including Src, caveolin-1, PLC, and IP3R, was increased, and NKA localization, which is altered in retinoschisin knockout mice (Friedrich et al., 2011) was restored in the photoreceptor inner segments (Plössl et al., 2017a). It has been further shown that ouabain can displace retinoschisin from NKA, though only at high µM to mM concentrations (Schmid et al., 2020). The authors of these studies proposed that retinoschisin normally binds to and suppresses NKA signaling in the retina, and that when retinoschisin is mutated or knocked out, NKA signaling is increased; this is demonstrated by decreased activations of Src and ERK in retinoschisin knockout cells in which the protein has been reintroduced (Plössl et al., 2017a). These results suggest a regulatory effect of retinoschisin on NKA signaling and reduction in retinoschisin expression could represent an initial step in XLRS pathogenesis.

### Bipolar disorder

Bipolar disorder (BD) is a genetic and highly debilitating mental health disorder characterized by extreme mood swings, including manic episodes of abnormally elevated mood alternating with depressive episodes. As in other conditions mentioned above, BD appears to be accompanied by abnormally high circulating levels of CTS. However, patients with BD have a specific increase of CTS within the parietal cortex (Goldstein et al., 2006), while the overall level of CTS in plasma is low (Grider et al., 1999; El-Mallakh et al., 2010). Interestingly, digitalis toxicity can be accompanied by manic-depressive symptoms in humans (Keller and Frishman, 2003). It has been shown that intracerebroventricular (ICV) injections of ouabain in rats induced mania and mimicked the manic phenotypes of bipolar disorder (El-Mallakh et al., 2003; Riegel et al., 2009; Sui et al., 2013; Logan and McClung, 2016). These effects were reduced using the common BD mood stabilizers, lithium and valproic acid (Brocardo et al., 2010; Varela et al., 2015). In addition, ICV injections of anti-ouabain antibodies lowered CTS concentrations in the brain, caused anti-depressive effects (Goldstein et al., 2006; Goldstein et al., 2012), and blocked the hyperactivation produced by amphetamine (Hodes et al., 2016; Hodes et al., 2018). ICV administration of ouabain in the frontal cortex, striatum, and hippocampus mediate these effects through classic NKA signaling pathways, including Akt and its substrates (Yu et al., 2010; Kim et al., 2013), as well as MEK and ERK (Kim et al., 2008). The increase of these second messengers was also seen after administration of the stimulant amphetamine and interestingly, anti-ouabain antibodies block the effects of amphetamine (Lichtstein et al., 2018). It is clear that the activation of downstream effectors of CTS-NKA interaction modifies neuronal activity and neurotransmission, altering behavior and inducing BD (El-Mallakh et al., 2022). Interestingly, injection of digoxin specific Fab fragments (Digibind), which antagonizes CTS effects, resulted in a transient significant beneficial effect on depression symptoms in BD patients (Zilberstein et al., 2023).

While the etiology of BD is unknown, numerous genes have been associated to this condition; among them is the NKA α3 isoform, in which expression is reduced in the prefrontal cortex of post-mortem patient brain samples (Tochigi et al., 2008). In agreement with NKA α3 playing a role in BD is the finding that the *Myshkin* mice, a genetic model carrying a missense inactivating mutation of NKA α3, shows a mood-related behavioral profile that is similar to that of bipolar patients in the manic state (Kirshenbaum et al., 2015). Moreover, like in the manic BD induced by ouabain, *Myshkin* mice show activation of Akt and ERK in the hippocampus, supporting the notion that the mechanism underlying this condition depends on either higher level of endogenous ouabain, or abnormally increased NKA α3 signaling. Interestingly, this could represent a case in which NKA has deficient pumping activity but hyperactivated signaling function.

## CTS and cancer

The role of NKA in cancer is a broad topic, which is outside the scope of this review; however, we would like to include a general view here. For a more detailed discussion of research in this area, we direct interested readers to several excellent reviews on the topic (Al-Ghoul and Valdes, 2008; Mijatovic et al., 2012; Slingerland et al., 2013; Alevizopoulos et al., 2014; Silva et al., 2021).

The idea of using CTS as anti-cancer agents developed from two main observations: one was the increase in NKA ion transport and activity that accompanies some type of cancer cells, and the other is the property of CTS to stimulate cell apoptosis. In addition, cancer cells show changes in expression of the NKA α and β subunits, alteration in the composition of its isoforms, and an increase in NKA sensitivity to CTS (Weidemann, 2005). Altogether, these data led to the idea of using NKA as a potential target for cancer therapy and a biomarker for cancer detection. However, studies correlating CTS use in cancer in patients gave conflicting results. While some studies observed that patients treated with digitalis have reduced cancer incidence and less recurrences of pre-existing cancers compared with patients not taking digitalis, others have shown no protective effects or a lack of improvement with respect to mortality (Al-Ghoul and Valdes, 2008; Osman et al., 2017; Zhang et al., 2017; Geng et al., 2020).

Early work showed that the CTS oleandrin decreased tumor development in skin cancer (Kanwal et al., 2020; Francischini et al., 2022). A variety of other cardenolides, including digitalis, digitoxin, hellebrin and ouabain have been shown to have similar effects in other cancer cell lines (Ayogu and Odoh, 2020). Moreover, besides natural compounds, some synthetic CTS have been tested for their use as anti-cancer agents (de Oliveira et al., 2021). While CTS present anti-proliferative effects on cancer cells, it is important to note that this is different from their growth effects in noncancerous cells. While not clear yet, it has been hypothesized that the difference in response depends on the pattern of NKA isoform expression or the dissimilar signaling pathways activated by CTS in normal versus cancer cells. It is important to note that most of the studies have tested the effect of different CTS on cells in culture, including breast, pancreatic, lung, prostate, melanoma, leukemia, neuroblastoma, and renal adenocarcinoma. In general, CTS mainly function on tumor cells as sensitizers of apoptosis and anoikis, or as inducers of autophagy-like death. For example, digoxin stimulated the activity of caspases 3/7 in gastric cancer cells, increasing anoikis and reducing the number of metastatic cells. This effect is mediated by the NKAα3 isoform (Numata et al., 2025). Digoxin also reduced circulating tumor cell clusters in patients with metastatic breast cancer (Kurzeder et al., 2025).

While the signaling function of NKA plays a role in cancer and exert some anti-cancer effect, the mechanisms of CTS action have not been completely elucidated. Among the various intracellular pathways that CTS activate, the most relevant to tumorigenesis is the downregulation of several transcription factors, such as c-Myc, NF-κB, and the hypoxia-inducible factor (HIF-1α), which are directly involved with cell growth, proliferation, and apoptosis (Mijatovic and Kiss, 2013). CTS downregulation of c-Myc also activates the expression of the multiple drug-resistant gene, which appears to be the mechanism by which CTS helps maintain the sensitivity of cancer cells to different drugs (Mijatovic and Kiss, 2013). The inhibition of HIF-1α production reduces the expression of proangiogenic genes, which will diminish blood flow to the tumor and consequently its development. CTS also upregulate the expression of the cell cycle inhibitor p21, inducing cell cycle arrest. Moreover, CTS have a radio sensitizing effect on malignant cancer cell lines, an event that depends on the downregulation of topoisomerases (Winnicka et al., 2006). Another pathway regulated by CTS is the PI3K-Akt pathway, which is involved in the activation of caspases and apoptosis of cancer cells (Yin et al., 2012). CTS have also been shown to solidify tight junctions in cancer cell, a mechanism that is important to reduce the metastatic nature of tumors (de Souza et al., 2014; Rocha et al., 2014). In addition, a loss of Na/K-ATPase-mediated Src regulation leads to stimulation of the Warburg phenotype in cancer, implying that that NKA signaling works as a suppressor of the abnormal metabolic changes in cancer cells (Banerjee et al., 2018). Recently, artificial intelligence, molecular docking and molecular dynamic simulations have been used to identify the mechanisms of action of bufalin. This approach predicted that the estrogen receptor (ESR1) is a potential target of bufalin. Further experimental analysis confirmed that bufalin interacts and stimulates degradation of ESR1. These data suggested that the anti-cancer effects of bufalin are related to its ability to overcome tumor endocrine resistance (Jiang et al., 2025).

It is important to point out that we are here providing a broad view of the actions of CTS. However, there are marked differences in the effect, potencies, and mechanism of actions of CTS and that this occurs in a cancer-type dependent manner. In addition, while the signaling role of NKA is important for the anti-cancer effect of CTS, it appears that the increase in intracellular calcium that results from the inhibition of NKA pumping also plays a role in inducing cancer cell apoptosis (Winnicka et al., 2006). In any case, targeting NKA with CTS has emerged as an attractive approach for the treatment of cancer. One issue with the use of CTS is the toxicity that these compounds present, which limits their use in the relatively high doses required to stop cell growth of cancer cells. Improving the therapeutic index, as well as enhancing the selectivity of effect of CTS, represent unmet goals which need to be achieved before they can be applied in cancer treatment.

## Conclusions and prospects

Following the captivating discovery of the signaling role of NKA as a receptor and a signal transduction molecule for CTS effects nearly 30 years ago, a significant amount of data and important progress has been made. However, as has happened with other dazing findings, the road has been challenging. The novel non-canonical role of NKA posed a number of questions and led to heated, controversial debates. While some points have been clarified, many others still remain to be elucidated. At earlier stages in the search for endogenous CTS, a point of disagreement revolved around the nature and origin of the circulating compounds. While mass spectrometry showed that ouabain itself or a ouabain isomer was the CTS agent present in blood, its endogenous nature was challenged. This was based on the fact that in mammals, steroids display the typical trans and not cis conformation, and that mammals do not have the capability to synthesize the rhamnose sugar present in ouabain. These criticisms have been justified, arguing that different components of the CTS molecule may come premade from exogenous sources. Unfortunately, our understanding of the biosynthetic pathways involved in the synthesis of ouabain are at present incomplete. While it is known that cholesterol is the main substrate for the synthesis of ouabain and that the first reaction steps are the cleavage of the side chain and 3β hydroxylation of cholesterol, the more distal reactions in the biosynthetic pathway are unknown (Lichtstein et al., 2012). Despite this, it seems clear that CTS, CTS-like substances and ouabain are present in body fluids; however, distinguishing their identity has been difficult. While the use of immunoassay techniques have shown the presence of CTS in plasma, the assumption that they correspond to ouabain has not always been confirmed by mass spectroscopy (Baecher et al., 2014). This shows that the various immunoassays developed to detect CTS may not yet have the selectivity needed to clearly distinguish between different cardenolides. Moreover, the difficulties in measuring the low levels of CTS in extracellular fluids, which showed high variability depending on the sample type and method used, has complicated their study. Despite these caveats, it is now well accepted that different CTS, including ouabain, are circulating in body fluids (Blaustein and Hamlyn, 2024), but more accurate and straightforward methods for CTS determination need to be developed.

Another point relevant to the effects of CTS has been the possibility that these compounds could be acting on a receptor different from the NKA. While several other targets of CTS have been suggested, evidence for a direct interaction of CTS with those targets have not been fully proven. Experiments in mice in which the ouabain affinity of NKA has been modified to alter their binding to ouabain show a good correlation between the binding capacity of NKA and CTS cell effects, suggesting that if not the only receptor, NKA is the main receptor for CTS (Lingrel, 2010). The affinity of the NKA for CTS varies depending on the species and NKA isoform considered. This raises new questions as to the role of different NKA isoforms in the response. In rodents, the differences in ouabain affinities of NKA isoforms (Blanco and Mercer, 1998), led to the assumption that in cells where various isoforms are expressed, the effect may be mediated by the isoform with the highest affinity for ouabain. However, in species different from rodents, the smaller differences in ouabain binding between isoforms makes it difficult to ascertain which NKA is mediating the effects. As mentioned above, the low ouabain affinity in rodents implies that NKA α1 would not be able to respond to the relatively low levels of CTS found in circulation. A mechanism that has been proposed to explain this inconsistency is the interaction among NKA αβ protomers that may allow occupation of a small fraction of NKA by CTS which may be sufficient to cause a large amplification of the downstream signaling effects (Liu and Askari, 2006; Orlov et al., 2021). While this topic merits further study, the current experimental evidence shows that, at physiologic levels, CTS can activate NKA signaling and it is possible that the differences in NKA ouabain affinity may have developed to differentially respond to these compounds in a cell specific manner (Takeyasu et al., 2001; Herbertz et al., 2024).

Another point regards the differences in effect of CTS, which vary depending on the amount of CTS used. While it is clear that relatively high doses of CTS inhibit NKA activity and low ones activate signaling, it has been reported that nanomolar or subnanomolar concentrations of ouabain can also stimulate NKA activity. This stimulation has been observed in whole homogenates prepared from rat brain, where the effect was ascribed to a displacement of an endogenous ouabain-like compound from NKA or changes induced by ouabain on the environment surrounding NKA (Lichtstein et al., 1985). An activation of NKA activity by ouabain was also reported in cardiac preparations, in which the effect appears to be mainly involve the NKA α2 isoform (Gao et al., 1999). However, these results remain unclear and further investigations are needed to find an explanation for this puzzling response.

Additional uncertainties over NKA signaling revolved around the underlying mechanism of NKA signaling, which to date is not completely clear. This particularly refers to the localization of the NKA signaling complex, which is unclear if it resides in only caveolae, the general cell plasma membrane, or in both. In addition, while there is consensus that Src is a key mediator of the downstream effects of ouabain, the way in which NKA cross talks with Src is still the matter of debate. Thus, there is evidence indicating a direct association between NKA and Src (Li and Xie, 2009) and using microscale thermophoresis, a dissociation constant (K_d_) for the NKA-Src complex was calculated to be about 0.2 µM (Petrushanko et al., 2022). On the other hand, other studies, using immunoprecipitation and blue native gels were unable to detect a stable Src-NKA complex (Li and Xie, 2009; Yosef et al., 2016). These differences may reflect the different type of preparations used for each study. In addition, it is unknown whether Src and NKA form a functional complex, or if their interaction is transient. Besides the upstream interactions of the NKA receptor, experimental evidence shows that downstream mechanisms of NKA signaling are dependent on the NKA isoform targeted by CTS and this may explain, at least in part, the cell specific actions of CTS (Madan et al., 2017; Yu et al., 2018). Overall, this highlights the complexity of mechanisms of action for NKA signaling and emphasizes the biological relevance of NKA isoforms.

Another interesting point is the difficulty in dissecting the effects of CTS that depend on the action on NKA ion transport from those resulting from the activation of NKA signaling function. The simultaneous action between those two roles of NKA and their crosstalk show the complex mechanisms that cells have developed to maintain their homeostasis and regulate their function. The multifaceted function of NKA, as well as the intriguing effects of CTS are strong drivers to continue investigating in this field of research. This will help us to develop pharmacologic approaches that, by interfering with CTS-induced and NKA-mediated signaling, or enhancing it, can be used to correct conditions in which this pathway is dysregulated.
